# Supplementary figures and images for: In vivo CRISPR/Cas9 knockout screen: TCEAL1 silencing enhances docetaxel efficacy in prostate cancer
Source: Life Sci Alliance. 2020 Oct 8;3(12):e202000770. doi: 10.26508/lsa.202000770 (PMC7556750; doi:10.26508/lsa.202000770)

Source Data - Figure 1

F.

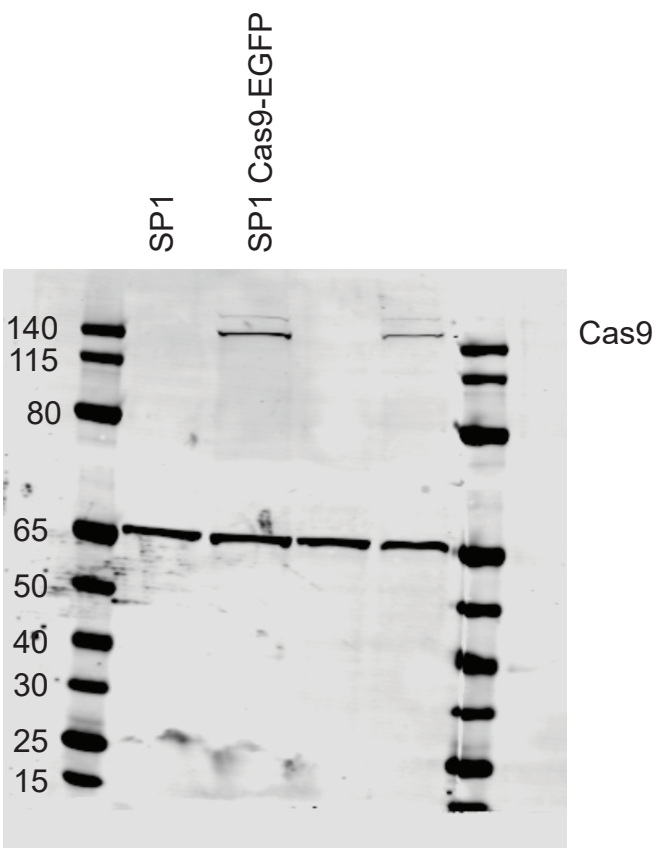

G.

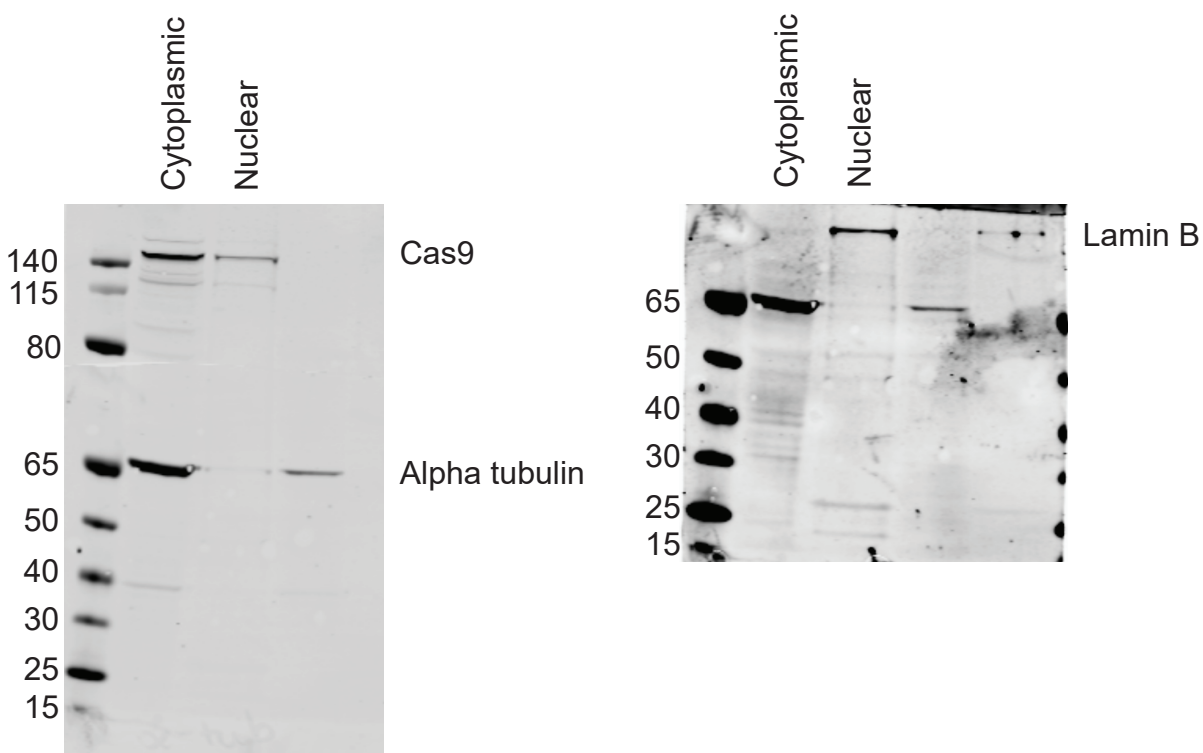

Supplement: Supplementary file 1 [file LSA-2020-00770_SdataF1.pdf]

Source Data - Figure 3

B.

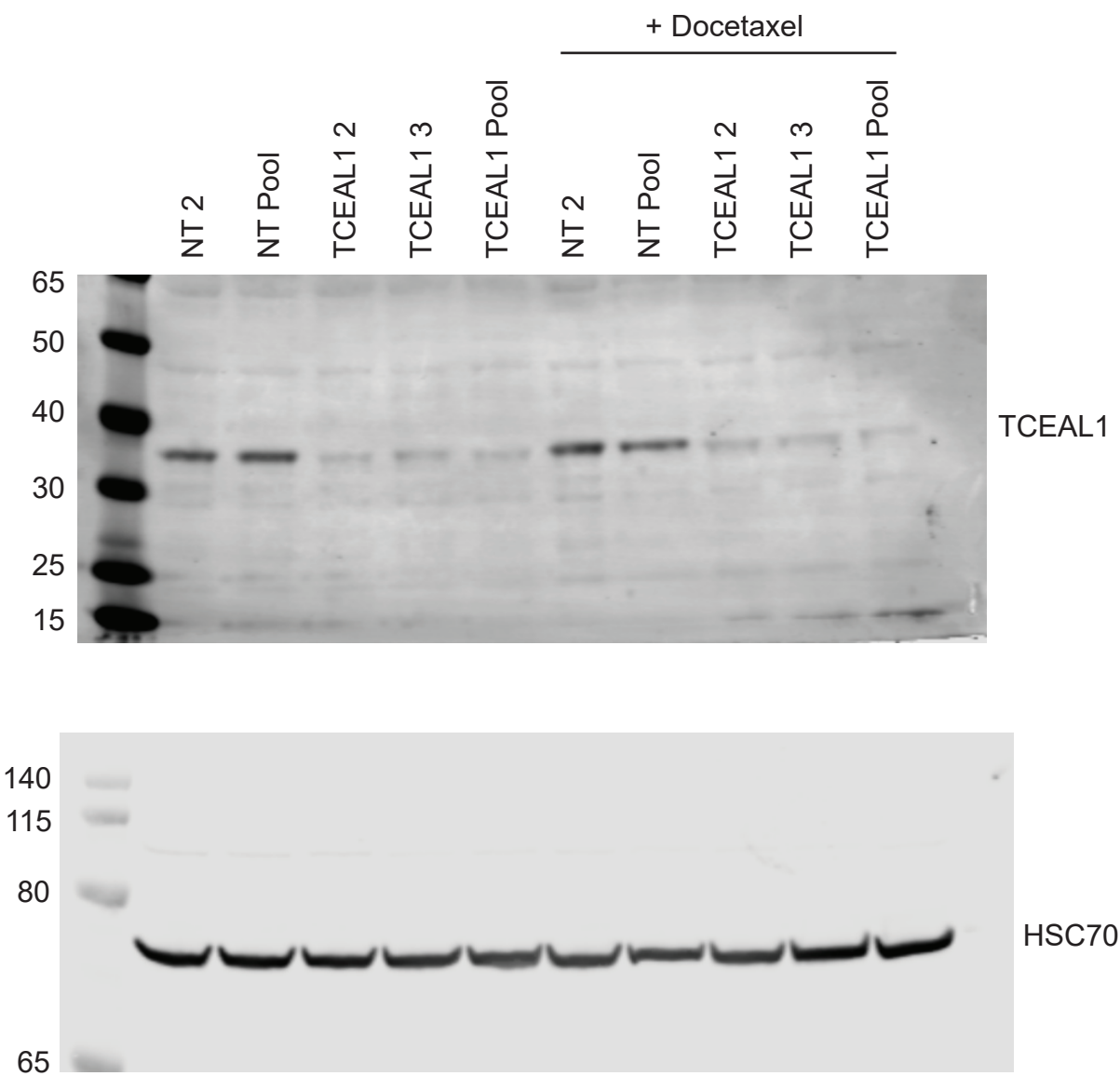

Supplement: Supplementary file 4 [file LSA-2020-00770_SdataF3.pdf]
