# Supplementary material for: In vivo CRISPR/Cas9 knockout screen: TCEAL1 silencing enhances docetaxel efficacy in prostate cancer
Source: Life Sci Alliance. 2020 Oct 8;3(12):e202000770. doi: 10.26508/lsa.202000770 (PMC7556750; doi:10.26508/lsa.202000770)
Supplement: Supplementary file 2 [file LSA-2020-00770_TableS1.docx]

**Supplementary Tables**

*In vivo* CRISPR/Cas9 knockout screen: TCEAL1 silencing enhances docetaxel efficacy in prostate cancer

**Table S1.** Overview of sequencing data

| **Condition** | **reads (millions)** | **mapped (millions)** | **mapped (%)** | **gini index** | **total sgRNAs** | **detected sgRNAs** | **detected sgRNAs (%)** | **total genes** | **detected genes** | **detected genes (%)** |
| --- | --- | --- | --- | --- | --- | --- | --- | --- | --- | --- |
| plasmid | 24.8 | 20.4 | 82.17 | 0.1148 | 65959 | 65374 | 99.11 | 21485 | 21485 | 100.00 |
| cells | 24.4 | 12.9 | 52.9 | 0.1351 | 65959 | 64902 | 98.40 | 21485 | 21484 | 100.00 |
| cells | 25.9 | 13.6 | 52.27 | 0.1339 | 65959 | 64902 | 98.40 | 21485 | 21484 | 100.00 |
| cells | 26.6 | 14.0 | 52.8 | 0.1332 | 65959 | 64902 | 98.40 | 21485 | 21484 | 100.00 |
| mock | 25.5 | 10.1 | 39.58 | 0.488 | 65959 | 47514 | 72.04 | 21485 | 20991 | 97.70 |
| mock | 25.1 | 20.0 | 79.63 | 0.6784 | 65959 | 27345 | 41.46 | 21485 | 17189 | 80.00 |
| mock | 22.2 | 13.3 | 59.88 | 0.648 | 65959 | 34434 | 52.21 | 21485 | 19077 | 88.79 |
| mock | 22.2 | 16.8 | 75.64 | 0.7374 | 65959 | 23174 | 35.13 | 21485 | 15556 | 72.40 |
| mock | 18.1 | 11.8 | 65.56 | 0.7365 | 65959 | 23484 | 35.60 | 21485 | 15721 | 73.17 |
| mock | 16.7 | 9.2 | 55.25 | 0.7558 | 65959 | 21648 | 32.82 | 21485 | 14834 | 69.04 |
| mock | 15.5 | 4.5 | 28.94 | 0.651 | 65959 | 29769 | 45.13 | 21485 | 17844 | 83.05 |
| mock | 20.7 | 14.9 | 72.01 | 0.6385 | 65959 | 30757 | 46.63 | 21485 | 18179 | 84.61 |
| mock | 28.4 | 18.6 | 65.4 | 0.6082 | 65959 | 33424 | 50.67 | 21485 | 18912 | 88.02 |
| docetaxel | 23.0 | 16.2 | 70.36 | 0.5198 | 65959 | 39018 | 59.15 | 21485 | 19987 | 93.03 |
| docetaxel | 24.6 | 22.4 | 91.12 | 0.7053 | 65959 | 29530 | 44.77 | 21485 | 17813 | 82.91 |
| docetaxel | 17.0 | 13.8 | 81.17 | 0.6581 | 65959 | 31692 | 48.05 | 21485 | 18444 | 85.85 |
| docetaxel | 24.5 | 20.2 | 82.45 | 0.6864 | 65959 | 27679 | 41.96 | 21485 | 17223 | 80.16 |
| docetaxel | 23.3 | 19.4 | 83.18 | 0.6734 | 65959 | 33616 | 50.96 | 21485 | 18943 | 88.17 |
