# Supplementary material for: In vivo CRISPR/Cas9 knockout screen: TCEAL1 silencing enhances docetaxel efficacy in prostate cancer
Source: Life Sci Alliance. 2020 Oct 8;3(12):e202000770. doi: 10.26508/lsa.202000770 (PMC7556750; doi:10.26508/lsa.202000770)
Supplement: Supplementary file 3 [file LSA-2020-00770_TableS2.docx]

**Supplementary Tables**

*In vivo* CRISPR/Cas9 knockout screen: TCEAL1 silencing enhances docetaxel efficacy in prostate cancer

**Table S2.** Sequences of TCEAL1 sgRNAs in GeCKOv2 library A and their predicted off target effects. Potential off-target effects for each of the three Tceal1 sgRNAs were examined using <https://wge.stemcell.sanger.ac.uk/find_off_targets_by_seq>. Two of the three TCEAL1 sgRNAs uniquely target TCEAL1. The third sgRNA could potentially target other genes, though with 3 or 4 mismatches. In our sequencing data however, MGLibA_53077 was only detected in Plasmid, Cells and one of the Vehicle samples, and so only the other two TCEAL1 sgRNAs were included in our vehicle and docetaxel comparison (shown in Table 2).

| sgRNA | sequence | WGE off targets results |
| --- | --- | --- |
| MGLibA_53075 | CGTATCCGCCCTCAATTCAT | https://wge.stemcell.sanger.ac.uk/crispr/565054205 |
| MGLibA_53076 | GTTCGAAGACCGTATTCCCA | https://wge.stemcell.sanger.ac.uk/crispr/565054187 |
| MGLibA_53077 | GTCTGAAGATCGTCCTCCGC | https://wge.stemcell.sanger.ac.uk/crispr/565054182 |
