# Supplementary material for: In vivo CRISPR/Cas9 knockout screen: TCEAL1 silencing enhances docetaxel efficacy in prostate cancer
Source: Life Sci Alliance. 2020 Oct 8;3(12):e202000770. doi: 10.26508/lsa.202000770 (PMC7556750; doi:10.26508/lsa.202000770)
Supplement: Supplementary file 5 [file LSA-2020-00770_TableS3.docx]

**Supplementary Tables**

*In vivo* CRISPR/Cas9 knockout screen: TCEAL1 silencing enhances docetaxel efficacy in prostate cancer

**Table S3.** Percentages (±SD) of PC3M cells in the indicated stages of the cell cycle (n=3 biological replicates). Percentages shown in red are increased compared to control, those in blue are decreased. Numbers in bold indicate the cell cycle stages that are most significantly altered for each treatment.

|  | **Control** | **Doc** | **TCEAL1 siRNA** | **TCEAL1 siRNA + Doc** |
| --- | --- | --- | --- | --- |
| **Sub G1** | 0.8 (±0.04) | **7.6 (±0.80)** | 2.9 (±0.84) | **11.5 (±1.82)** |
| **G1** | 49.1 (±0.78) | **25.5 (±1.47)** | **41.0 (±1.17)** | 31.3 (±2.60) |
| **S** | 8.7 (±0.47) | 13.8 (±1.93) | 10.7 (±0.35) | 13.2 (±1.93) |
| **G2/M** | 37.5 (±1.35) | 44.4 (±1.93) | 38.8 (±0.47) | 32.8 (±2.47) |
| **Polyploidy** | 3.9 (±0.12) | 8.4 (±0.35) | 6.9 (±0.97) | **11.8 (±1.16)** |
