# Supplementary material for: In vivo CRISPR/Cas9 knockout screen: TCEAL1 silencing enhances docetaxel efficacy in prostate cancer
Source: Life Sci Alliance. 2020 Oct 8;3(12):e202000770. doi: 10.26508/lsa.202000770 (PMC7556750; doi:10.26508/lsa.202000770)
Supplement: Supplementary file 6 [file LSA-2020-00770_TableS4.docx]

**Supplementary Tables**

*In vivo* CRISPR/Cas9 knockout screen: TCEAL1 silencing enhances docetaxel efficacy in prostate cancer

**Table S4.** Expression of MDR genes following TCEAL1 knockdown. TCEAL1 knockdown only resulted in small degree of changes in the MDR gene expression, ranging from -1.57 to +1.34. Of note, ABCB7 is the only down regulated gene showing statistical significance following TCEAL1 knockdown. Among the upregulated MDR encoding genes as a result of TCEAL1 silencing, upregulation of ABCB9 and ABCB10 expression (borderline increase at 1.21 and 1.34 fold respectively) would suggest that the enhanced response was unrelated to the MDR expression following TCEAL1 knockdown.

| **Gene** | **Mean TCEAL1 siRNA** | **Mean Control siRNA** | **Adjusted p value** | **Fold Change** |
| --- | --- | --- | --- | --- |
| *ABCB1* | 1 | 3 | #N/A | -1.20 |
| *ABCB4* | 11 | 8 | 0.643237006 | 1.16 |
| *ABCB6* | 104 | 109 | 0.843738072 | -1.04 |
| *ABCB7* | 1535 | 2428 | 6.61924E-10 | -1.57 |
| *ABCB8* | 1653 | 1848 | 0.083587956 | -1.12 |
| *ABCB9* | 508 | 420 | 0.008957489 | 1.21 |
| *ABCB10* | 931 | 673 | 0.044105831 | 1.34 |
| *ABCB11* | 46 | 46 | 0.97878136 | 1.01 |
