# Supplementary material for: In vivo CRISPR/Cas9 knockout screen: TCEAL1 silencing enhances docetaxel efficacy in prostate cancer
Source: Life Sci Alliance. 2020 Oct 8;3(12):e202000770. doi: 10.26508/lsa.202000770 (PMC7556750; doi:10.26508/lsa.202000770)
Supplement: Supplementary file 7 [file LSA-2020-00770_TableS5.docx]

**Supplementary Tables**

*In vivo* CRISPR/Cas9 knockout screen: TCEAL1 silencing enhances docetaxel efficacy in prostate cancer

**Table S5.** Statistics

| **Figure** | **Test** | **Comparison** | **p Value** |
| --- | --- | --- | --- |
| 1B | Mann-Whitney | *Pten^-/-^ V Pten^-/-^ Spry2^-/+^* | 0.0303 |
| 1C | Log-rank Mantel-Cox | Vehicle V Docetaxel | 0.0065 |
| 1D | Mann-Whitney | Vehicle V Docetaxel | 0.0159 |
| 1I | Mann-Whitney | Vehicle V Docetaxel | 0.3826 |
| 2D | 1-way ANOVA with Tukey's | SP1 - Control V Doc | <0.0001 |
|  |  | SP1 - Control V Tceal1 siRNA | 0.9869 |
|  |  | SP1 - Control V Tceal1 siRNA + Doc | <0.0001 |
|  |  | SP1 - Doc V Tceal1 siRNA + Doc | 0.0319 |
|  |  | SP1 - Tceal1 siRNA V Tceal1 siRNA + Doc | <0.0001 |
|  |  | PC3M - Control V Doc | 0.0076 |
|  |  | PC3M - Control V TCEAL1 siRNA | 0.0273 |
|  |  | PC3M - Control V TCEAL1 siRNA + Doc | <0.0001 |
|  |  | PC3M - Doc V TCEAL1 siRNA + Doc | 0.01 |
|  |  | PC3M - TCEAL1 siRNA V TCEAL1 siRNA + Doc | 0.003 |
|  |  | CWR22 - Control V Doc | 0.0017 |
|  |  | CWR22 - Control V TCEAL1 siRNA | 0.0112 |
|  |  | CWR22 - Control V TCEAL1 siRNA + Doc | <0.0001 |
|  |  | CWR22 - Doc V TCEAL1 siRNA + Doc | 0.019 |
|  |  | CWR22 - TCEAL1 siRNA V TCEAL1 siRNA + Doc | 0.0027 |
|  |  | DU145 - Control V Doc | <0.0001 |
|  |  | DU145 - Control V TCEAL1 siRNA | 0.9022 |
|  |  | DU145 - Control V TCEAL1 siRNA + Doc | <0.0001 |
|  |  | DU145 - Doc V TCEAL1 siRNA + Doc | 0.0012 |
|  |  | DU145 - TCEAL1 siRNA V TCEAL1 siRNA + Doc | <0.0001 |
|  |  | LNCaP - Control V Doc | 0.0004 |
|  |  | LNCaP - Control V TCEAL1 siRNA | 0.04 |
|  |  | LNCaP - Control V TCEAL1 siRNA + Doc | <0.0001 |
|  |  | LNCaP - Doc V TCEAL1 siRNA + Doc | 0.0267 |
|  |  | LNCaP - TCEAL1 siRNA V TCEAL1 siRNA + Doc | 0.0003 |
| 3A | 2-way ANOVA with Sidak's | NT2 V NT pool | 0.9713 |
|  |  | NT2 V TCEAL1 2 | 0.0112 |
|  |  | NT2 V TCEAL1 3 | 0.0008 |
|  |  | NT2 V TCEAL1 pool | 0.0093 |
| 3C | 1-way ANOVA with Tukey's | RWPE - Control V Doc | 0.0001 |
|  |  | RWPE - Control V TCEAL1 siRNA | 0.6726 |
|  |  | RWPE - Control V TCEAL1 siRNA + Doc | <0.0001 |
|  |  | RWPE - Doc V TCEAL1 siRNA + Doc | 0.9998 |
|  |  | RWPE - TCEAL1 siRNA V TCEAL1 siRNA + Doc | <0.0001 |
| 3E | 1-way ANOVA with Tukey's | SubG1 - Control V Doc | <0.0001 |
|  |  | SubG1 - Control V Tceal1 siRNA | 0.2637 |
|  |  | SubG1 - Control V Tceal1 siRNA + Doc | <0.0001 |
|  |  | SubG1 - Doc V Tceal1 siRNA + Doc | 0.0083 |
|  |  | SubG1 - Tceal1 siRNA V Tceal1 siRNA + Doc | <0.0001 |
|  |  | G1 - Control V Doc | <0.0001 |
|  |  | G1 - Control V TCEAL1 siRNA | <0.0001 |
|  |  | G1 - Control V TCEAL1 siRNA + Doc | <0.0001 |
|  |  | G1 - Doc V TCEAL1 siRNA + Doc | <0.0001 |
|  |  | G1 - TCEAL1 siRNA V TCEAL1 siRNA + Doc | <0.0001 |
|  |  | S - Control V Doc | 0.0003 |
|  |  | S - Control V TCEAL1 siRNA | 0.3168 |
|  |  | S - Control V TCEAL1 siRNA + Doc | 0.0016 |
|  |  | S - Doc V TCEAL1 siRNA + Doc | 0.9524 |
|  |  | S - TCEAL1 siRNA V TCEAL1 siRNA + Doc | 0.1354 |
|  |  | G2M - Control V Doc | <0.0001 |
|  |  | G2M - Control V TCEAL1 siRNA | 0.65 |
|  |  | G2M - Control V TCEAL1 siRNA + Doc | 0.0011 |
|  |  | G2M - Doc V TCEAL1 siRNA + Doc | <0.0001 |
|  |  | G2M - TCEAL1 siRNA V TCEAL1 siRNA + Doc | <0.0001 |
|  |  | Polyploidy - Control V Doc | 0.0015 |
|  |  | Polyploidy - Control V TCEAL1 siRNA | 0.0564 |
|  |  | Polyploidy - Control V TCEAL1 siRNA + Doc | <0.0001 |
|  |  | Polyploidy - Doc V TCEAL1 siRNA + Doc | 0.0285 |
|  |  | Polyploidy - TCEAL1 siRNA V TCEAL1 siRNA + Doc | 0.0006 |
| 4D | 1-way ANOVA with Tukey's | CDC25A - Control V Doc | 0.5904 |
|  |  | CDC25A - Control V TCEAL1 siRNA | 0.0228 |
|  |  | CDC25A - Control V TCEAL1 siRNA + Doc | 0.0008 |
|  |  | CDC25A - Doc V TCEAL1 siRNA + Doc | 0.0034 |
|  |  | CDC25A - TCEAL1 siRNA V TCEAL1 siRNA + Doc | 0.0918 |
|  |  | CHEK1 - Control V Doc | 0.5185 |
|  |  | CHEK1 - Control V TCEAL1 siRNA | 0.0181 |
|  |  | CHEK1 - Control V TCEAL1 siRNA + Doc | 0.0013 |
|  |  | CHEK1 - Doc V TCEAL1 siRNA + Doc | 0.0069 |
|  |  | CHEK1 - TCEAL1 siRNA V TCEAL1 siRNA + Doc | 0.2133 |
|  |  | ESPL1 - Control V Doc | 0.5403 |
|  |  | ESPL1 - Control V TCEAL1 siRNA | 0.0086 |
|  |  | ESPL1 - Control V TCEAL1 siRNA + Doc | 0.0064 |
|  |  | ESPL1 - Doc V TCEAL1 siRNA + Doc | 0.0405 |
|  |  | ESPL1 - TCEAL1 siRNA V TCEAL1 siRNA + Doc | 0.9949 |
|  |  | DSCC1 - Control V Doc | 0.9746 |
|  |  | DSCC1 - Control V TCEAL1 siRNA | 0.0052 |
|  |  | DSCC1 - Control V TCEAL1 siRNA + Doc | 0.0044 |
|  |  | DSCC1 - Doc V TCEAL1 siRNA + Doc | 0.0074 |
|  |  | DSCC1 - TCEAL1 siRNA V TCEAL1 siRNA + Doc | 0.9991 |
| EV2D | 1-way ANOVA with Tukey's | Cul9 - Control V Doc | 0.0049 |
|  |  | Cul9 - Control V Tceal1 siRNA | 0.0735 |
|  |  | Cul9 - Control V Tceal1 siRNA + Doc | 0.0002 |
|  |  | Cul9 - Doc V Tceal1 siRNA + Doc | 0.0454 |
|  |  | Cul9 - Tceal1 siRNA V Tceal1 siRNA + Doc | 0.0033 |
|  |  | Wdr72 - Control V Doc | <0.0001 |
|  |  | Wdr72 - Control V TCEAL1 siRNA | 0.0004 |
|  |  | Wdr72 - Control V TCEAL1 siRNA + Doc | <0.0001 |
|  |  | Wdr72 - Doc V TCEAL1 siRNA + Doc | <0.0001 |
|  |  | Wdr72 - TCEAL1 siRNA V TCEAL1 siRNA + Doc | <0.0001 |
| EV2E | 1-way ANOVA with Tukey's | CUL9 - Control V Doc | 0.0007 |
|  |  | CUL9 - Control V Tceal1 siRNA | 0.0023 |
|  |  | CUL9 - Control V Tceal1 siRNA + Doc | <0.0001 |
|  |  | CUL9 - Doc V Tceal1 siRNA + Doc | 0.0007 |
|  |  | CUL9 - Tceal1 siRNA V Tceal1 siRNA + Doc | 0.0002 |
|  |  | WDR72 - Control V Doc | 0.0007 |
|  |  | WDR72 - Control V TCEAL1 siRNA | <0.0001 |
|  |  | WDR72 - Control V TCEAL1 siRNA + Doc | <0.0001 |
|  |  | WDR72 - Doc V TCEAL1 siRNA + Doc | <0.0001 |
|  |  | WDR72 - TCEAL1 siRNA V TCEAL1 siRNA + Doc | 0.0011 |
| EV3B | 1-way ANOVA with Dunnett's | NT2 V NT pool | 0.9779 |
|  |  | NT2 V TCEAL1 2 | 0.0785 |
|  |  | NT2 V TCEAL1 3 | 0.0009 |
|  |  | NT2 V TCEAL1 pool | 0.0137 |
| EV4B | 1-way ANOVA with Tukey's | MMS22L - Control V Doc | 0.9117 |
|  |  | MMS22L - Control V TCEAL1 siRNA | 0.0110 |
|  |  | MMS22L - Control V TCEAL1 siRNA + Doc | <0.0001 |
|  |  | MMS22L - Doc V TCEAL1 siRNA + Doc | <0.0001 |
|  |  | MMS22L - TCEAL1 siRNA V TCEAL1 siRNA + Doc | 0.0033 |
|  |  | PLK4 - Control V Doc | 0.1950 |
|  |  | PLK4 - Control V TCEAL1 siRNA | 0.0027 |
|  |  | PLK4 - Control V TCEAL1 siRNA + Doc | 0.0006 |
|  |  | PLK4 - Doc V TCEAL1 siRNA + Doc | 0.0077 |
|  |  | PLK4 - TCEAL1 siRNA V TCEAL1 siRNA + Doc | 0.5473 |
|  |  | E2F2 - Control V Doc | 0.6548 |
|  |  | E2F2 - Control V TCEAL1 siRNA | <0.0001 |
|  |  | E2F2 - Control V TCEAL1 siRNA + Doc | <0.0001 |
|  |  | E2F2 - Doc V TCEAL1 siRNA + Doc | <0.0001 |
|  |  | E2F2 - TCEAL1 siRNA V TCEAL1 siRNA + Doc | 0.0371 |
|  |  | E2F8 - Control V Doc | 0.3097 |
|  |  | E2F8 - Control V TCEAL1 siRNA | 0.0007 |
|  |  | E2F8 - Control V TCEAL1 siRNA + Doc | 0.0002 |
|  |  | E2F8 - Doc V TCEAL1 siRNA + Doc | 0.0010 |
|  |  | E2F8 - TCEAL1 siRNA V TCEAL1 siRNA + Doc | 0.4724 |
